# *PC* Gene Affects Milk Production Traits in Dairy Cattle

**DOI:** 10.3390/genes15060708

**Published:** 2024-05-29

**Authors:** Aixia Du, Zijiao Guo, Ao Chen, Lingna Xu, Dongxiao Sun, Bo Han

**Affiliations:** Department of Animal Genetics and Breeding, College of Animal Science and Technology, Key Laboratory of Animal Genetics, National Engineering Laboratory for Animal Breeding, State Key Laboratory of Animal Biotech Breeding, China Agricultural University, Breeding and Reproduction of Ministry of Agriculture and Rural Affairs, Beijing 100193, China; duaixia@cau.edu.cn (A.D.); gzjiao@cau.edu.cn (Z.G.); capudrooks@foxmail.com (A.C.); xulingna@caas.cn (L.X.); sundx@cau.edu.cn (D.S.)

**Keywords:** *PC* gene, single-nucleotide polymorphism, milk production trait, genomic selection, transcription factor

## Abstract

In previous work, we found that *PC* was differentially expressed in cows at different lactation stages. Thus, we deemed that *PC* may be a candidate gene affecting milk production traits in dairy cattle. In this study, we found the polymorphisms of *PC* by resequencing and verified their genetic associations with milk production traits by using an animal model in a cattle population. In total, we detected six single-nucleotide polymorphisms (SNPs) in *PC*. The single marker association analysis showed that all SNPs were significantly associated with the five milk production traits (*p* < 0.05). Additionally, we predicted that allele G of 29:g.44965658 in the 5′ regulatory region created binding sites for TF GATA1 and verified that this allele inhibited the transcriptional activity of *PC* by the dual-luciferase reporter assay. In conclusion, we proved that *PC* had a prominent genetic effect on milk production traits, and six SNPs with prominent genetic effects could be used as markers for genomic selection (GS) in dairy cattle, which is beneficial for accelerating the improvement in milk yield and quality in Chinese Holstein cows.

## 1. Introduction

Milk is a kind of nutrition-rich food, often as a family daily diet health food of advanced nutrition. As the main component of milk, protein and fat play a crucial role in the evaluation of milk quality, in that its content directly determines the nutritional value of milk. Milk fat is a quality ingredient for butter and cheese, which contains unsaturated fatty acid in preventing atherosclerosis. Milk protein is necessary to convert milk into cheese and other milk [1]. Through a large number of research experiments and theoretical reviews, it is found that the main factors affecting milk production traits in cows is breed, meaning the genetics and breeding, accounting for more than 40%, have an outstanding contribution to the improvement in the production efficiency of the dairy cow breeding industry. Its most important economic traits are milk production traits, including milk, fat and protein yields and fat and protein percentages, in dairy cow breeding [2]. Milk production traits are controlled by multiple genes and affected by various factors including genetics, nutrition and the environment, thus making dairy cow breeding very difficult [3].

Genomic selection (GS) can reflect the issue of minor genes for quantitative traits [4,5,6,7]. Since the implementation of the genomic assessment in 2009, the genetic gain of US Holstein bulls has increased by 79.49% (milk), 151.55% (fat) and 192.25% (protein), and the generation interval has shortened to 2.2 years [8]. In 2012, GS began to be applied and popularized in China, which has also achieved a remarkable effect in dairy cows. High-throughput single-nucleotide polymorphism (SNP) marker genotyping is the premise of GS. Moreover, finding the functional site information with large genetic effects on the target trait and applying it to the chip can help us to better improve the accuracy of GS [9,10,11]. Based on this, researchers are continuously exploring genes and loci related to important complex traits.

Previously, we studied the proteomes of the liver in three Chinese Holstein cows from three periods—dry period (50 days before lactation), early lactation (10 days after lactation) and peak lactation (60 days after lactation)—and found that the pyruvate carboxylase (*PC*) gene that was enriched in the metabolic pathways related to milk synthesis such as the lipid metabolic process and pyruvate metabolism was significantly highly expressed in early lactation compared to the other two periods (dry period vs. early lactation: fold change = 0.63, *p* = 0.000000465; early vs. peak lactation: fold change = 1.32, *p* = 0.001926) [12]. The *PC* gene encodes pyruvate carboxylase, an important metabolic enzyme, which leads to the carboxylation of pyruvate into oxaloacetate with the participation of biotin and ATP [13]. *PC* is involved in many metabolic reactions, such as gluconeogenesis, lipogenesis, insulin secretion and the synthesis of the neurotransmitter glutamate [14]. It plays a crucial role in the process of glucose production in the liver, and its expression increases during the perinatal period to accommodate the increased glucose requirement [15,16]. In addition, seven SNPs, identified in previous reports, rs109496284, rs110561408, rs137492467, rs42194999, rs42195000, rs42195007 and rs42197370, in the *PC* gene (Chr.29:44862572–44965356; Cattle Quantitative Trait Locus Database) were significantly associated with the milk protein percentage, and these SNPs were located QTL regions for milk yield (QTL_ID: 2593, 4506) [17] and protein yield (QTL_ID:2612) [18]. Therefore, we inferred that the *PC* gene might be a vital functional gene influencing the milk production traits of cows.

Herein, we detected SNPs of the *PC* gene and analyzed their genetic associations with milk production traits. In addition, we predicted the potential effects of confirmed SNPs on the transcription factor binding site (TFBS) and checked the effect of the SNP at the 5′ regulatory region on the transcriptional activity of the *PC* gene by the dual-luciferase reporter assay, then conjecturing the causal mutation of milk production traits in cattle.

## 2. Materials and Methods

### 2.1. Animals and Phenotypic Data

We used 925 Chinese Holstein cows which were from 44 sire families for association analyses, and these cows were spread in 21 dairy farms which belong to the Beijing Shounong Animal Husbandry Development Co., Ltd. (Beijing, China). These cows, with identical feeding conditions, were well and had precise pedigree information as well as normative dairy herd improvement (DHI) records. The descriptive statistics of the phenotypic number for dairy production traits are shown in Table S1.

### 2.2. DNA Extraction

The samples were provided by Beijing Dairy Cattle Center. DNAs were extracted from frozen semen by the salt-out procedure and blood samples by a TIANamp Blood DNA Kit (Tiangen, Beijing, China), respectively. Next, the NanoDrop 2000 Spectrophotometer (Thermo Scientific, Hudson, NH, USA) and gel electrophoresis were used to judge the quantity and quality of the extracted DNAs.

### 2.3. SNP Identification and Genotyping

We designed 36 primers (Table S2) in the *PC* gene’s parts of the intron region, coding region and 2000 bp of upstream and downstream regions by Primers3 (https://primer3.ut.ee/, accessed on 8 January 2024). We amplified the semen DNAs which were mixed equally by PCR (Table S2) and used gel electrophoresis to perceive the PCR amplification products before Sanger sequencing by BGI. We identified the potential SNPs in the light of the reference sequences (ARS-UCD1.2) on NCBI-BLAST (https://blast.ncbi.nlm.nih.gov/Blast.cgi, accessed on 16 February 2024) after sequencing. In the aftermath, we genotyped the six SNPs in 925 cows by the Genotyping by Target Sequencing (GBTS) technology of Boruidi Biotechnology Co., Ltd. (Shijiazhuang, China).

### 2.4. Association Analyses

The MIXED process in SAS 9.4 software was used to conduct association analyses between the SNPs and the five milk production traits, which included 305-day milk yield, fat yield, fat percentage, protein yield and protein percentage, on each lactation. The additive genetic relationship matrix A or the kinship matrix was computed by tracing the pedigree back three generations to 2761 involved individuals. Our animal model for using association analysis was as follows:

y=μ+HYS+b×M+G+a+e

where **y** is the phenotypic value for each trait; **µ** is the overall mean; **HYS** is the fixed effect of herd (1~21 for 21 herds, separately), year (1~4 for the year 2012~2015, separately) and season (1 for April~May; 2 for June~August; 3 for September~November; and 4 for December~March, separately); **M** is a covariant of the age of calving with 21 levels; **b** is the regression coefficient of covariant **M**; **G** is the genotype combination effect; a is the individual random additive genetic effect, distributed as $N(0,A\delta_{a}^{2})$, with the additive genetic variance $\delta_{a}^{2}$; and **e** is the random residual, distributed as $N\left(0,I\delta_{e}^{2}\right)$, with identity matrix I and residual error variance $\delta_{e}^{2}$.

In addition, we figured out the additive effect (a), dominant effect (d) and substitution effect (α) by the following equations:

$$a=\frac{AA-BB}{2},\;d=AB-\frac{AA+BB}{2},\;\alpha =a+d(q-p)$$

where AA, BB and AB are the least square means of the milk production traits in the corresponding genotypes, p is the frequency of allele A and q is the frequency of allele B.

### 2.5. Functional Prediction and Verification of Mutation Sites in 5′ Region of PC

We predicted transformations of the TFBSs for the SNP located in the 5′ region of *PC* by JASPAR (https://jaspar.genereg.net/, accessed on 10 March 2024, relative score > 0.90).

Further, we verified the effect of the SNP site on the gene expression activity by the dual-luciferase reporter assay. For 29:g.44965658G>A, we constructed the fragment with a SNP site, G or A, and cloned the fragments carrying the endonuclease sites KpnI and Nhel, respectively, into the pGL 4.14 luciferase reporter vector (Promega, Madison, WI, USA). The integrity of each insert was confirmed by sequencing the constructed plasmids. The plasmids were extracted for cell transfection by the Endo-free Plasmid Maxi Kit (Omega Bio-tek, Inc., Norcross, GA, USA). Human embryonic kidney 293T cells were cultured with 10% fetal bovine serum (FBS; Gibco) before transfection. Then, the cells were transiently transfected with Lipofectamine 2000 (Thermo Scientific, Beijing, China). It was co-transfected with 500 ng of the constructed plasmid and 10 ng of pRL-TK Renilla luciferase reporter vector (Promega) in each well. The cells were harvested for luciferase activity detection by a Dual-Luciferase Reporter Assay System (Promega) after a transfection of 48 h. The relative fluorescence activity was calculated by the fluorescence activity ratio of firefly and Renilla.

## 3. Results

### 3.1. SNPs Identification

**Figure 1:**
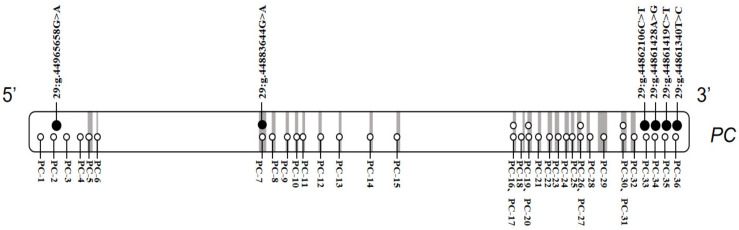
Position of SNPs and primers in *PC* gene. Gray boxes represent exons. Solid black circles represent SNPs. Hollow black circles represent primers.

In this study, we found six SNPs in total in the *PC* gene. One SNP, 29:g.44965658G>A, was located in the 5′ regulatory region, one SNP, a synonymous mutation, 29:g.44883644G>A, in exon 3, and four SNPs, 29:g.44862106C>T, 29:g.44861428A>G, 29:g.44861419C>T and 29:g.44861340T>C, in the 3′ regulatory region (Table 1 and Figure 1). The information of all the identified SNPs is summarized in Table 1.

### 3.2. Associations between SNPs and Five Milk Production Traits

We analyzed the associations between the six SNPs in the *PC* gene and milk production traits in cattle. In the first lactation, there were two, five, five and one SNPs significantly associated with milk, fat and protein yields and protein percentage, respectively (*p* < 0.05). One SNP, 29:g.44965658G>A, had significant genetic association with milk, fat, protein yields and protein percentage (*p* < 0.05). The SNP 29:g.44883644G>A was significantly associated with fat yield (*p* < 0.05). Three SNPs, 29:g.44862106C>T, 29:g.44861428A>G and 29:g.44861340T>C, were significantly associated with fat and protein yields (*p* < 0.05). The SNP 29:g.44861419C>T had significant genetic association with milk and protein yields (*p* < 0.05; Table 2). In the second lactation, there were four, four, four and three SNPs that were associated with milk yield, fat yield, fat percentage and protein yield (*p* < 0.05), respectively. One SNP, 29:g.44965658G>A, had significant genetic association with milk and protein yields (*p* < 0.01). The SNP 29:g.44883644G>A was significantly associated with fat yield and percentage (*p* < 0.05). Three SNPs, 29:g.44862106C>T, 29:g.44861428A>G and 29:g.44861340T>C, were significantly associated with milk and fat yields and fat percentage (*p* < 0.05). Two SNPs, 29:g.44862106C>T and 29:g.44861419C>T, had significant genetic association with protein yield (*p* < 0.05; Table 2). In both lactations, the SNP 29:g.44965658G>A had a significant genetic effect on milk and protein yields (*p* < 0.01; Table 2). In addition, the results of other allelic effects of the SNPs in the *PC* gene are displayed in Table S3.

### 3.3. The Regulation of the 5′ Region SNPs on Transcriptional Activity

We used JASPAR software (https://jaspar.genereg.net/, accessed on 10 March 2024) to predict the changes in TFBSs caused by the SNP 29:g.44965658G>A on the 5′ region of the *PC* gene and found that the allele G of 29:g.44965658 created binding sites (BSs) for transcription factor (TF) GATA1 (score = 0.90).

**Figure 2:**
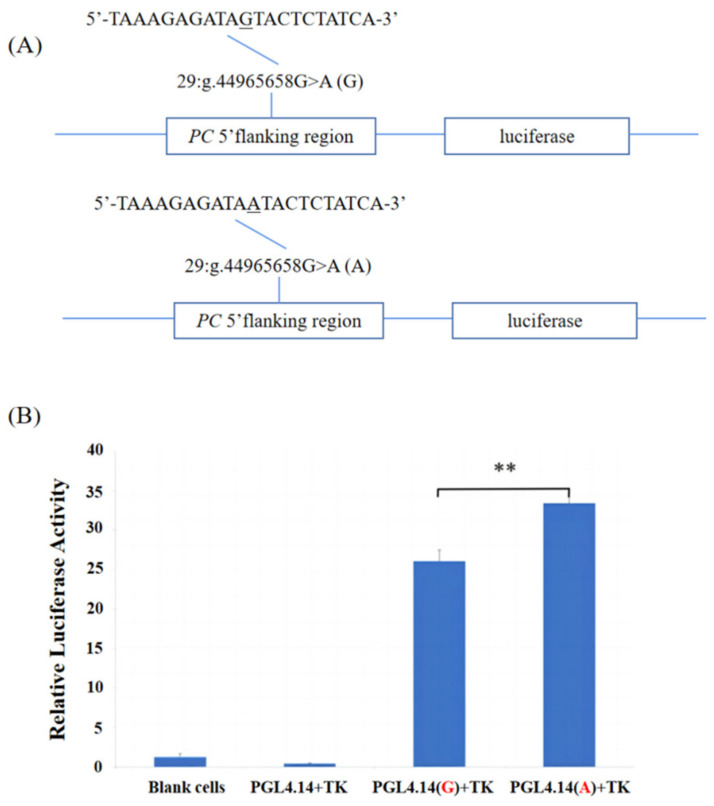
Dual-luciferase activity assay. (**A**) Sketches of recombinant plasmids with 29:g.44965658G>A in the *PC* gene. The underlined nucleotide was the SNP. (**B**) A luciferase activity analysis of the recombinant plasmids in HEK 293T cells. ** *p* < 0.01. The red font represents the SNP(29:g.44965658G>A) allele.

Subsequently, we instituted reporter plasmids containing two alleles G and A (Figure 2A), respectively, to further determine whether the SNP, 29:g.44965658G>A, in the 5′ regulatory region varied the transcription activity of the *PC* gene. As shown in Figure 2B, we found that the inserted fragments have a transcriptional regulation function by comparing the luciferase activity of the two recombinant plasmids and the empty vector and the blank cell controls (*p* < 0.01). The luciferase activity of the A allele was significantly higher than that of the G allele (*p* < 0.01), indicating that the transcriptional activity of the *PC* gene was significantly increased after the allele mutation.

## 4. Discussion

A previous study showed that the *PC* gene may be a candidate gene for influencing milk production traits in cattle [12]. We discovered the polymorphisms of *PC* and sought out that there was a notable genetic association between the SNPs and five milk production traits. Studies have shown that it is more accurate and less biased to predict traits when we give SNPs different weights according to the importance of SNPs in the genomic relationship matrices’ traits [10,19]. Thus, the significant SNPs we found could be applied to GS, and it helped to speed up the breeding process of dairy cows, improved the annual average genetic progress of milk production traits and obtained higher milk yield and quality. Currently, six SNPs of the *PC* gene in this study are not present in four gene chips (GeneSeek Genomic Profiler (GGP) Bovine 150 K and 100 K arrays, illumina Bovine SNP50K BeadChip, illumina BovineHD Genotyping BeadChip), and then we can add these SNPs which are given different weights based on their effect on milk production traits in a commercial SNP chip to improve the accuracy of genomic prediction.

Transcription factors (TFs) are a group of protein molecules that can ensure a specific intensity expression of the target gene at a specific time and space when they bind to TFBSs [20]. When the SNP site is located at TFBSs, it will affect the binding of transcription factors, leading to differential gene expression between individuals of different genotypes [21,22,23]. In this study, the SNP, 29:g.44965658G>A, in the 5′ regulatory region of *PC* was predicted and verified to change the TFBSs that would impact the expression of the *PC* gene. The mutation from allele G to A of 29:g.44965658G>A led to the disappearance of TFBSs for TF GATA1. The co-binding of TAL1/SCL to GATA1 could play a role in gene repression by recruiting co-repressors [24,25,26]. The transcription factor GATA-1 formed repressive complexes GATA-1/Gfi-1b and GATA-1/FOG-1/MeCP1 that suppressed the genes *GATA2*, *MYC* and *MYB* involved in cell proliferation [27]. Therefore, we speculate that transcription factor GATA1 binding to the G site of 29:g.44965658G>A may repress the expression of the *PC* gene. Furthermore, with the dual-luciferase assay, we found that the transcriptional activity of the *PC* gene was significantly increased when G was mutated to A in 29:g.44965658G>A, suggesting that *PC* gene expression might be inhibited by TF GATA1 via binding the G site. *PC* is an important anaplerotic enzyme that replenishes the tricarboxylic acid cycle (TCA) intermediates [28]. It has been shown that the *PC* gene has quantitative trait loci (QTL: 2593, 2612, 255006) that are very close, and this locus has a significant effect on milk yield, protein yield and percentage [18,29]. The expression of the *PC* gene influences metabolic processes in milk synthesis, and when the expression of the *PC* gene is inhibited, it may promote milk production traits [30]. In summary, the allele G of 29: G.44965658G>A can bind to TF GATA1, leading to the inhibition of *PC* gene expression and ultimately affecting milk production traits in dairy cows.

## 5. Conclusions

In summary, we confirmed six SNPs in the *PC* gene and confirmed their genetic effect on milk production traits in Chinese Holstein cows. The SNP 29:g.44965658G>A may be the crucial mutation site for milk production traits, possibly regulating the transcriptional activity of the *PC* gene by binding transcription factors, and the specific mechanism needs to be further verified. This study lays the foundation for a further validation of the function of *PC* in milk synthesis, where its valuable SNPs can be used as candidate markers for dairy cow molecular breeding for the development of a GS customized chip.

## Figures and Tables

**Table 1:** Details of SNPs identified in *PC* gene.

SNP Name	GenBank No.	Location	Genotype	Genotypic Frequency	Allele	Allelic Frequency
29:g.44965658G>A	rs42193753	5′ regulatory region	GG	0.0908	G	0.3103
			AG	0.4389	A	0.6897
			AA	0.4703		
29:g.44883644G>A	rs42195007	exon 3	GG	0.0303	G	0.1773
			AG	0.2941	A	0.8227
			AA	0.6757		
29:g.44862106C>T	rs110381742	3′ regulatory region	CC	0.7038	C	0.8405
			CT	0.2735	T	0.1595
			TT	0.0227		
29:g.44861428A>G	rs109519857	3′ regulatory region	AA	0.7038	A	0.8405
			AG	0.2735	G	0.1595
			GG	0.0227		
29:g.44861419C>T	rs133706500	3′ regulatory region	CC	0.3838	C	0.6211
			CT	0.4746	T	0.3789
			TT	0.1416		
29:g.44861340T>C	rs110386158	3′ regulatory region	TT	0.7049	T	0.8411
			CT	0.2724	C	0.1589
			CC	0.0227		

**Table 2:** Associations of six SNPs in *PC* with milk production traits in two lactations of Chinese Holstein cows (LSM ± SE).

SNP Name	Lactation	Genotype (No.)	Milk Yield (kg)	Fat Yield (kg)	Fat Percentage (%)	Protein Yield (kg)	Protein Percentage (%)
29:g.44965658G>A	1	GG (84)	10,053 ^A^ ± 188.17	325.58 ± 7.89	3.26 ± 0.08	302.03 ^A^ ± 5.75	3.02 ^A^ ± 0.05
		AG (406)	10,051 ^A^ ± 177.94	326.14 ^A^ ± 7.54	3.27 ± 0.07	297.18 ^B^ ± 5.5	2.97 ^B^ ± 0.05
		AA (435)	9876.16 ^B^ ± 178.56	320.52 ^B^ ± 7.57	3.27 ± 0.07	293.6 ^C^ ± 5.52	2.99 ± 0.05
		*P*	0.001	0.0089	0.8672	0.0005	0.0165
	2	GG (62)	10,415 ^A^ ± 112.87	373.96 ^A^ ± 4.69	3.62 ± 0.05	311.14 ^A^ ± 3.42	3 ± 0.03
		AG (245)	10,692 ^B^ ± 70.11	385.1 ^B^ ± 3.04	3.61 ± 0.03	315.1 ^A^ ± 2.21	2.96 ± 0.02
		AA (296)	10,792 ^B^ ± 64.48	384.72 ^B^ ± 2.83	3.58 ± 0.03	319.99 ^B^ ± 2.06	2.97 ± 0.02
		*P*	0.004	0.0509	0.4316	0.007	0.3234
29:g.44883644G>A	1	GG (28)	10,178 ± 221.89	339.1 ^A^ ± 9.11	3.35 ± 0.09	303.76 ± 6.64	3.00 ± 0.05
		AG (272)	10,038 ± 179.99	324.44 ^B^ ± 7.62	3.26 ± 0.07	296.84 ± 5.55	2.97 ± 0.05
		AA (625)	9971.64 ± 176.75	324.5 ^B^ ± 7.5	3.27 ± 0.07	296.95 ± 5.47	2.99 ± 0.05
		*P*	0.1752	0.0146	0.1773	0.1743	0.1503
	2	GG (23)	10,499 ± 174.8	357.71 ^A^ ± 7.14	3.44 ^A^ ± 0.07	315.89 ± 5.21	3.02 ± 0.04
		AG (173)	10,635 ± 77.47	378.92 ^B^ ± 3.31	3.58 ± 0.03	314.64 ± 2.41	2.97 ± 0.02
		AA (407)	10,756 ± 60.82	387.01 ^C^ ± 2.69	3.61 ^B^ ± 0.07	318.3 ± 1.96	2.97 ± 0.02
		*P*	0.1313	<0.0001	0.0425	0.2506	0.5181
29:g.44862106C>T	1	CC (651)	9987.86 ^A^ ± 176.71	324.89 ^A^ ± 7.5	3.27 ± 0.07	297.34 ^A^ ± 5.47	2.99 ± 0.05
		CT (253)	10,016 ^A^ ± 180.39	323.46 ^A^ ± 7.63	3.25 ± 0.07	296.19 ^A^ ± 5.56	2.97 ± 0.05
		TT (21)	10,337 ^B^ ± 234.65	339.82 ^B^ ± 9.57	3.30 ± 0.09	307.37 ^B^ ± 6.98	2.99 ± 0.06
		*P*	0.0705	0.0209	0.4895	0.0331	0.2059
	2	CC (427)	10,781 ^A^ ± 60.26	387.89 ^A^ ± 2.67	3.61 ^A^ ± 0.02	318.81 ^A^ ± 1.95	2.97 ± 0.02
		CT (159)	10,548 ^B^ ± 80.12	375.88 ^B^ ± 3.41	3.58 ^A^ ± 0.03	313.44 ^B^ ± 2.48	2.98 ± 0.02
		TT (17)	10,495 ± 203.88	353.35^C^ ± 8.29	3.4 ^B^ ± 0.08	311.29 ± 6.05	2.98 ± 0.05
		*P*	0.0064	<0.0001	0.0314	0.0402	0.7269
29:g.44861428A>G	1	AA (651)	9983.94 ^A^ ± 176.7	324.84 ^A^ ± 7.5	3.27 ± 0.07	297.21 ^A^ ± 5.47	2.99 ± 0.05
		AG (253)	10,028 ^A^ ± 180.4	323.62 ^A^ ± 7.63	3.25 ± 0.07	296.56 ^A^ ± 5.56	2.97 ± 0.05
		GG (21)	10,339 ^B^ ± 234.65	339.86 ^B^ ± 9.57	3.3 ± 0.09	307.45 ^B^ ± 6.98	2.99 ± 0.06
		*P*	0.058	0.0224	0.4197	0.0407	0.2204
	2	AA (428)	10,775 ± 60.22 ^A^	387.92 ± 2.67 ^A^	3.61 ± 0.02 ^A^	318.67 ± 1.94 ^A^	2.97 ± 0.02
		AG (158)	10,562 ± 80.28 ^B^	375.71 ± 3.41 ^B^	3.57 ± 0.03 ^A^	313.78 ± 2.49 ^B^	2.98 ± 0.02
		GG (17)	10,496 ± 203.89	353.29 ± 8.29 ^C^	3.4 ± 0.08 ^B^	311.32 ± 6.05	2.98 ± 0.05
		*P*	0.0137	<0.0001	0.0225	0.0647	0.7735
29:g.44861419C>T	1	CC (355)	9898.04 ^A^ ± 179.13	322.33 ± 7.59	3.28 ± 0.07	294.45 ^A^ ± 5.53	2.99 ± 0.05
		CT (439)	10,002 ^B^ ± 177.59	324.18 ± 7.53	3.27 ± 0.07	296.72 ± 5.49	2.98 ± 0.05
		TT (131)	10,089 ^B^ ± 183.81	326.08 ± 7.74	3.25 ± 0.07	300.11 ^B^ ± 5.64	2.99 ± 0.05
		*P*	0.0168	0.3576	0.6528	0.0166	0.7129
	2	CC (245)	10,786 ^A^ ± 68.41	385.42 ± 2.97	3.59 ± 0.03	319.68 ^A^ ± 2.17	2.97 ± 0.02
		CT (273)	10,697 ± 67.39	382.81 ± 2.94	3.59 ± 0.03	316.81 ± 2.14	2.97 ± 0.02
		TT (85)	10,553 ^B^ ± 97.13	381 ± 4.07	3.62 ± 0.04	311.51 ^B^ ± 2.96	2.96 ± 0.02
		*P*	0.0623	0.4761	0.7483	0.0217	0.8088
29:g.44861340T>C	1	TT (652)	9986.08 ^A^ ± 176.7	324.9 ^A^ ± 7.5	3.27 ± 0.07	297.3 ^A^ ± 5.47	2.99 ± 0.05
		CT (252)	10,022 ^A^ ± 180.43	323.43 ^A^ ± 7.63	3.25 ± 0.07	296.31 ^A^ ± 5.56	2.97 ± 0.05
		CC (21)	10,338 ^B^ ± 234.65	339.82 ^B^ ± 9.57	3.3 ± 0.09	307.39 ^B^ ± 6.98	2.99 ± 0.06
		*P*	0.0652	0.0207	0.422	0.0359	0.1949
	2	TT (428)	10,775 ^A^ ± 60.22	387.92 ^A^ ± 2.67	3.61 ^A^ ± 0.02	318.67 ^A^ ± 1.94	2.97 ± 0.02
		CT (158)	10,562 ^B^ ± 80.28	375.71 ^B^ ± 3.41	3.57 ^A^ ± 0.03	313.78 ^B^ ± 2.49	2.98 ± 0.02
		CC (17)	10,496 ± 203.89	353.29 ^C^ ± 8.29	3.4 ^B^ ± 0.08	311.32 ± 6.05	2.98 ± 0.05
		*P*	0.0137	<0.0001	0.0225	0.0647	0.7735

LSM ± SE: least squares mean ± standard deviation; the number in the bracket represents the number of cattle for the corresponding genotype; *P* shows the significance for the genetic effects of SNPs; A, B, C within the same column with different superscripts means *p* < 0.05.

## Data Availability

The original contributions presented in the study are included in the article and Supplementary Material, further inquiries can be directed to the corresponding author.

## Supplementary Materials

The following supporting information can be downloaded at https://www.mdpi.com/article/10.3390/genes15060708/s1, Table S1: Descriptive statistics of phenotypic values for the dairy production traits of the first and second lactations; Table S2: Information for PCR amplification; Table S3: Additive, dominant and allele substitution effects of six SNPs on milk production traits of *PC* gene in Chinese Holstein.
